# Preclinical Evidences for an Antimanic Effect of Carvedilol

**DOI:** 10.1155/2015/692541

**Published:** 2015-05-14

**Authors:** Greicy Coelho de Souza, Julia Ariana de S. Gomes, Ana Isabelle de Góis Queiroz, Maíra Morais de Araújo, Lígia Menezes Cavalcante, Michel de Jesus Souza Machado, Aline Santos Monte, David Freitas de Lucena, João Quevedo, André Ferrer Carvalho, Danielle Macêdo

**Affiliations:** ^1^Neuropharmacology Laboratory, Department of Physiology and Pharmacology, Faculty of Medicine, Federal University of Ceará, Rua Coronel Nunes de Melo 1127, 60431-270 Fortaleza, CE, Brazil; ^2^Laboratório de Neurociências, Programa de Pós-Graduação em Ciências da Saúde, Unidade Acadêmica de Ciências da Saúde, Universidade do Extremo Sul Catarinense, 88806-000 Criciúma, SC, Brazil; ^3^Center for Experimental Models in Psychiatry, Department of Psychiatry and Behavioral Sciences, The University of Texas Medical School at Houston, Houston, TX 77030, USA; ^4^Psychiatry Research Group, Faculty of Medicine, Federal University of Ceará, 60430-160 Fortaleza, CE, Brazil

## Abstract

Oxidative imbalance, alterations in brain-derived neurotrophic factor (BDNF), and mitochondrial dysfunction are implicated in bipolar disorder (BD) pathophysiology and comorbidities, for example, cardiovascular conditions. Carvedilol (CVD), a nonselective beta-blocker widely used for the treatment of hypertension, presents antioxidant and mitochondrial stabilizing properties. Thus, we hypothesized that CVD would prevent and/or reverse mania-like behavioral and neurochemical alterations induced by lisdexamfetamine dimesylate (LDX). To do this, male Wistar rats were submitted to two different protocols, namely, prevention and reversal. In the prevention treatment the rats received daily oral administration (mg/kg) of CVD (2.5, 5 or 7.5), saline, valproate (VAL200), or the combination of CVD5 + VAL100 for 7 days. From the 8th to 14th day LDX was added. In the reversal protocol LDX was administered for 7 days with the drugs being added from the 8th to 14th day of treatment. Two hours after the last administration the behavioral (open field and social interaction) and neurochemical (reduced glutathione, lipid peroxidation, and BDNF) determinations were performed. The results showed that CVD prevented and reversed the behavioral and neurochemical alterations induced by LDX. The administration of CVD5 + VAL100 potentiated the effect of VAL200 alone. Taken together these results demonstrate a possible antimanic effect of CVD in this preclinical model.

## 1. Introduction

Bipolar disorder (BD) is one of the most serious mental illnesses characterized by depressive and manic episodes with spontaneous cycling. The pathophysiology of this mental disorder remains unclear; however, evidences point towards the involvement of genetics, signal transmission deregulation, deleterious inflammatory profile, dysregulation in oxidative stress, and neurotrophins [1, 2].

The disorder presents a chronic course associated with functional decline, elevated mortality, and significant disease burden [3, 4]. Importantly, BD patients present an excess burden of cardiovascular risk and higher rate of hypertension compared to the general population [4]. Additionally, this risk is increased by modern treatments for BD, such as antipsychotics [5].

The number of affective episodes in BD patients is directly related to social and cognitive deficits as well as risk of suicide among others [6]. Still in relation to affective episodes, the number of manic episodes was related to impairment of verbal memory implicating, thus, frontal structures [3]. Besides frontal structures, such as prefrontal cortex, striatum and hippocampus are putative brain areas related to mania as observed in clinical [7] and preclinical studies [8, 9].

Based on the importance of manic episodes for BD outcome [10], the preclinical model most widely used to study BD is based on the induction of mania-like episodes by the administration of amphetamine-related compounds, such as d-amphetamine (d-AMPH) [11, 12]. Due to the restrictions on d-AMPH acquisition and use, recently our research group proposed an animal model of mania in rats based on the administration of lisdexamfetamine dimesylate (LDX) [9], a prodrug metabolically converted to d-AMPH [13]. In our previous study [9], LDX caused behavioral and neurochemical alterations similar to those of d-AMPH [11, 14] with the alterations prevented and reversed by the mood stabilizer lithium.

Indeed, the administration of amphetamine-related compounds to rodents resembles some alterations related to mania [1] such as increase in dopamine (DA) neurotransmission with consequent hyperlocomotion, oxidative imbalance, and mitochondrial dysfunction, and decrease in brain derived neurotrophic factor (BDNF) in putative brain areas related to BD pathophysiology, namely, prefrontal cortex (PFC), hippocampus (HC), and striatum (ST) [9, 14, 15].

Thus, based on the prooxidant alterations and mitochondrial dysfunctions observed in BD patients [1, 5] and in the animal models of mania [9, 15] as well as on the cardiovascular risk presented by BD patients, we decided to study the effects of carvedilol (CVD, {1-(carbazolyl-(4)-oxy)-3-(2-methoxyphenoxyethyl amino)-propanol-(2)}) against mania induced by LDX.

Carvedilol is prescribed for the treatment of congestive heart failure, mild to moderate hypertension, and myocardial infarction. The drug competitively blocks *β*1, *β*2, and *α*1-adrenergic receptors while displaying vasodilating properties. A distinctive characteristic of CVD in comparison to other *β*-adrenergic receptor antagonists is its potent antioxidant properties [16]. This antioxidant activity of CVD is attributed to its ability to chelate free iron [17]. The drug also presents mitochondria protective [18] and antiapoptotic/anti-inflammatory properties [19].

Therefore, herein we aimed to determine the effects of CVD alone and associated with the mood stabilizer drug, valproate, in the prevention and/or reversal of behavioral (hyperlocomotion and social interaction) and neurochemical (reduced glutathione (GSH) and lipid peroxidation) alterations in the PFC and ST, as well as hippocampal BDNF levels of animals submitted to the model of mania induced by LDX [9].

## 2. Materials and Methods

### 2.1. Drugs

Carvedilol (CVD; Coreg, Roche, Brazil), lisdexamfetamine dimesylate (LDX; Vyvanse, Shire, USA), and sodium valproate (VAL; Life Pharmaceutical Company) were used. The drugs were made up freshly within 1-2 h of dosing. All other chemicals used were of analytical grade.

### 2.2. Animals

The experiments were performed in adult male Wistar rats (weighting: 180–250 g) obtained from the Animal House of Federal University of Ceará. The animals were housed 6 per cage in standard polycarbonate rat cages (42 × 20.5 × 20 cm) and standard environmental conditions (22 ± 1°C; humidity 60 ± 5%; 12 h light/dark cycle with lights on at 7:00 am) with access to food (FRI-LAB Rat II, FRI-Ribe) and water* ad libitum*. All experimental procedures were conducted between 8:00 and 14:00 h and were carried out in accordance with the NIH Guide for the Care and Use of Laboratory Animals [20] and the Brazilian College of Animal Experimentation (COBEA). The raters were blind to the experimental groups. This research protocol was approved by the local ethical committee of Federal University of Ceará.

### 2.3. Study Design

**Figure 1:**
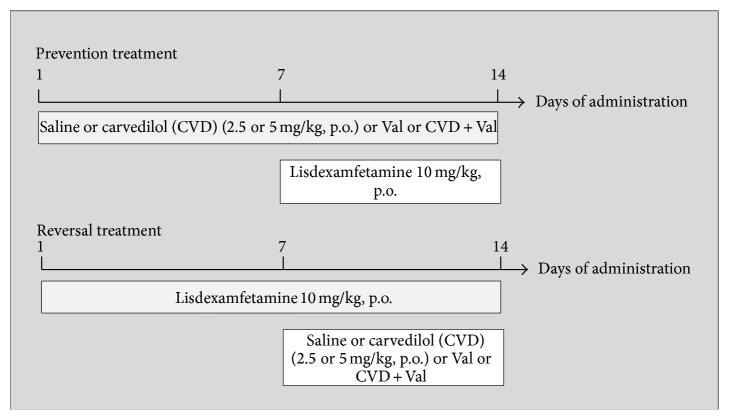
Schematic representation of the experimental design.

The rats were randomly divided into experimental groups (8–10 animals/group) distributed in two protocols, namely, prevention and reversal treatments (Figure 1), as described below. The use of LDX 10 mg/kg, p.o., to induce a mania-like behavior was based on a previous study from our research group [9]. The doses of CVD used here were calculated based on previous studies showing the neuroprotective effects of this drug [21] and human doses used for hypertension treatment as described elsewhere [22].

#### 2.3.1. Prevention Treatment

In the prevention model, we simulated the maintenance phase of BD treatment, as previously proposed [23]. Briefly, different groups of animals were treated with CVD (2.5, 5, or 7.5 mg/kg, p.o.) once a day, VAL (200 mg/kg i.p.) twice a day, the association of VAL 100 mg/kg, i.p. (twice a day) + CVD 5 mg/kg, with a 15 min interval between drugs in the first daily administration of VAL, or saline for 14 days. The reason for choosing the association CVD 5 mg/kg + VAL 100 mg/kg was based, in the case of CVD, on the first behavioral results obtained where it seemed for us that this was the best dose to conduct this protocol; in the case of VAL the dose was reduced by half to determine a possible potentiation of its effect by CVD. It is important to mention that the dose of VAL usually used in models of mania is 200 mg/kg twice a day [11]. Between the 8th and 14th day, the experimental groups additionally received one oral dose of LDX daily. The time interval between the administration of the drugs and LDX was 30 min.

Locomotor activity using the open field test (as described in Section 2.4) and social interaction (as described in Section 2.5) were measured on the 14th day of drug administration, 2 hours after the last drug administration. Following behavioral determinations, the rats were sacrificed by decapitation and the prefrontal cortex (PFC), hippocampus (HC), and striatum (ST) were dissected, rapidly frozen, and stored at −70°C until assayed.

#### 2.3.2. Reversal Treatment

In the reversal model, we reproduced the treatment of acute manic episodes as previously proposed [9]. In brief, each group of animals received one oral daily dose of LDX (10 mg/kg) or saline for 14 days. On the 8th day of treatment, the animals in the saline and LDX group additionally received oral administration of CVD (2.5, 5, and 7.5 mg/kg) once a day, VAL (200 mg/kg) twice a day, the association of VAL 100 mg/kg i.p. (twice a day) + CVD (with a 15 min interval), or saline, with a 1-hour interval between treatments. Locomotor activity and social interaction were measured on the 14th day of treatment, 2 hours after the last drug administration. After behavioral determinations the rats were sacrificed and the brain areas dissected for the neurochemical determinations.

In the present study our primary outcome was to determine the behavioral changes induced by CVD alone and associated with VAL in the model of mania induced by LDX. The secondary outcome was to determine the neurochemical alterations underlying these alterations.

### 2.4. Open Field Test

The locomotor activity was assessed using the open field test [24]. This test was performed in a 50 × 50 cm open field surrounded by 50 cm high walls made of acrylic. The open field floor was divided into four equal parts by black lines. The apparatus was placed in a red light room. The animals were gently placed on the center of the field and allowed to freely explore the arena for 5 min. Crossings of the black lines (used to determine horizontal activity) and rearing behavior (used to determine vertical activity) were counted, during the 5 min period, by experienced raters who were blinded to treatment.

### 2.5. Social Interaction Test

The testing apparatus consisted of a 60 × 40 cm Plexiglas box divided into three chambers. Rats were able to move between chambers through a small opening (6 × 6 cm) in the dividers. Iron cages in each of the two side chambers contained, in one side, the probe rat, whereas in the other side, the cage was empty. Test animals were placed in the center chamber. Rats were allowed 5 min of exploration time in the box, after which an unfamiliar, same-sex probe rat from the same experimental group was placed in one of two restraining cages [25]. The time spent in each of the three chambers was measured, and social preference was defined as follows: (% time spent in the social chamber) − (% time spent in the opposite chamber).

### 2.6. Neurochemical Determinations

#### 2.6.1. Tissue Preparation

Brain tissue samples were homogenized (10 times (w/v) with ice-cold 0.1 M phosphate buffer (pH 7.4). The homogenates were centrifuged at 10,000 rpm for 15 minutes, and aliquots of supernatants were separated and used for determination of oxidative stress parameters.

For enzyme immunoassay determinations (ELISA) 20 times (w/v) homogenates prepared in cold phosphate-buffered saline (PBS, pH 7.4) were used. A protease inhibitor cocktail (Sigma-Aldrich, St. Louis, USA) was added to the buffer and the homogenate was centrifuged at 14,000 rpm for 30 min.

#### 2.6.2. Determination of Reduced Glutathione (GSH) Levels

Reduced glutathione levels were evaluated to estimate endogenous defenses against oxidative stress. The method was based on Ellman's reagent (DTNB) reaction with free thiol groups [26]. The brain areas were diluted in EDTA 0.02 M buffer (10% w/v) and added to a 50% trichloroacetic acid solution. After centrifugation (3,000 rpm/15 min), the supernatant of the homogenate was collected and mixed with 0.4 M tris-HCl buffer, pH 8.9, and 0.01 M 5,5-dithiobis 2-nitrobenzoic acid (DTNB). The yellow color product was read immediately at 412 nm using a spectrophotometer (Beckman coulter UV/Visible). Results were calculated based on a standard glutathione curve and are expressed as ng of GSH/g wet tissue.

#### 2.6.3. Thiobarbituric Acid Reactive Species (TBARS) Levels

Lipid peroxides formation was analyzed by measuring the thiobarbituric-acid reacting substances (TBARS) in the homogenates [27] as an index of reactive oxygen species (ROS) production. The samples were mixed with 1 mL of trichloroacetic acid 10% (TCA) and 1 mL of thiobarbituric acid 0.67% (TBA), then heated in a boiling water bath for 15 min, and immediately kept cold in a bath of ice. Lipid peroxidation was determined by the absorbance at 532 nm. Results are expressed as *μ*mol of malonaldehyde (MDA)/g tissue.

#### 2.6.4. Determination of Hippocampal BDNF Levels

The levels of BDNF (ELISA; Millipore, USA) were determined in each sample by enzyme immunoassays according to the specific manufacturers' directions. Results are expressed as pg/g tissue.

### 2.7. Statistical Analysis

Statistical analyses were performed with GraphPad Prism 6.0 for Windows, GraphPad Software (San Diego, CA, USA). The results of the behavioral and neurochemical studies are expressed as means ± SEM (standard errors of the mean). Regular two-way ANOVA with “treatment protocol” and “experimental groups” as factors was performed. Tukey's test was used as post hoc test. Before ANOVA, D'Agostino-Pearson omnibus test was conducted to verify the normal distribution of the data. For all analyses, the significance level was set at *α* = 0.05.

## 3. Results

### 3.1. Carvedilol Alone and Associated with VAL Prevented and Reversed Hyperlocomotion and Alterations in Social Interaction Induced by LDX

**Figure 2:**
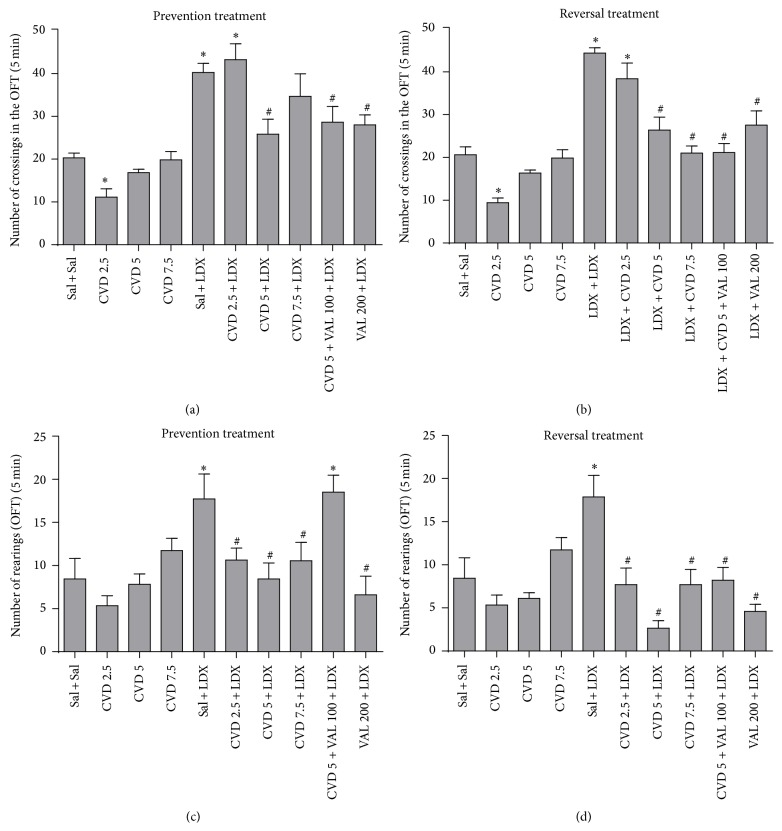
Number of crossings and rearings in the open field test in animals submitted to the prevention ((a), (c)) and reversal ((b), (d)) treatments. Data were analyzed by one-way ANOVA followed by Student-Newman-Keuls post hoc test. Values represent mean ± SEM (6–8 animals/group). ^∗^ *P* < 0.05 versus Sal + Sal; ^#^ *P* < 0.05 versus Sal + LDX or LDX + LDX. CVD: carvedilol; LDX: lisdexamfetamine; OFT: open field test; Sal: saline; VAL: valproate.

Locomotor agitation and increased sociability are common features of mania [28]. In the present study two-way ANOVA of the number of crossings revealed a significant interaction between “treatment protocol” and “experimental group” [*F*_9,88_ = 2.287, *P* = 0.0425] with significant main effect of “treatment protocol” [*F*_1,88_ = 4.210, *P* = 0.0431] and “experimental group” [*F*_9,88_ = 21.02, *P* < 0.001]. Regarding rearing behavior there was no significant interaction between factors [*F*_9,85_ = 1.57, *P* = 0.1671], but significant main effects of “treatment protocol” [*F*_1,85_ = 10.02, *P* = 0.0022] and “experimental groups” [*F*_9,85_ = 9.77, *P* < 0.0001] were observed. Post hoc analysis showed that in both prevention and reversal protocols LDX caused hyperlocomotion (Figures 2(a) and 2(b)) and increased rearing behavior (Figures 2(c) and 2(d)) as compared to control (Sal + Sal) rats. In the prevention treatment the administration of CVD 5, the combination of CVD5 + VAL100, and VAL200 significantly prevented the hyperlocomotion induced by LDX (*P* < 0.001). Regarding rearing behavior CVD 2.5, CVD5, CVD7.5, and VAL200 prevented the increase in this parameter (*P* < 0.001). In the reversal treatment CVD5, CVD7.5, CVD5 + VAL100, and VAL200 significantly reversed the hyperlocomotion induced by LDX (*P* < 0.001). The increase in rearing behavior was reversed by CVD 2.5, CVD5, CVD7.5, CVD5 + VAL100, and VAL200 (*P* < 0.001). After the administration of CVD alone we could observe that only CVD 2.5 significantly decreased the number of crossings in both treatments.

**Figure 3:**
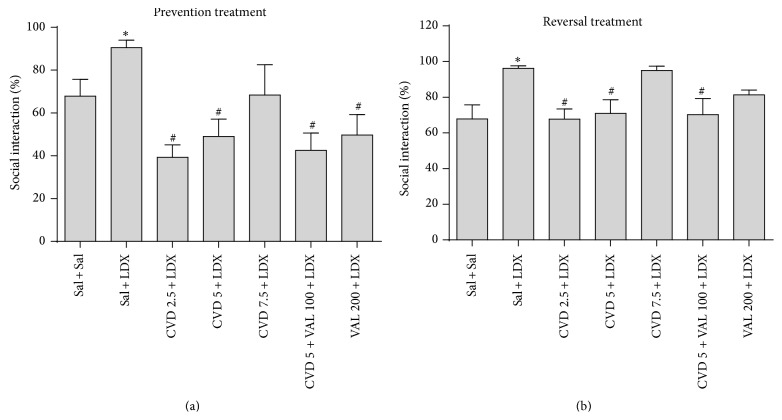
Percent of social interaction in animals submitted to the prevention (a) and reversal (b) treatments. Data were analyzed by one-way ANOVA followed by Student-Newman-Keuls post hoc test. Values are expressed as mean ± SEM (6–8 animals/group). ^∗^ *P* < 0.05 versus Sal + Sal; ^#^ *P* < 0.05 versus Sal + LDX or LDX + LDX. CVD: carvedilol; LDX: lisdexamfetamine; Sal: saline; VAL: valproate.

In the evaluation of social interaction two-way ANOVA revealed a significant interaction between factors [*F*_6,72_ = 2.24, *P* = 0.0490] with significant main effect of “treatment protocol” [*F*_1,72_ = 19.48, *P* < 0.0001] and “experimental group” [*F*_6,72_ = 5.28, *P* = 0.0001]. Post hoc analysis showed that the administration of LDX in both prevention and reversal treatments significantly increased the percent of social interaction as compared to control (Sal + Sal) animals. In the prevention paradigm CVD2.5, CVD5, CVD5 + VAL100, and VAL200 significantly prevented the alteration induced by LDX (*P* < 0.01) (Figure 3(a)). On the other hand in the reversal paradigm CVD2.5, CVD5, and CVD5 + VAL100 significantly reversed the alterations induced by LDX (*P* < 0.05) (Figure 3(b)).

### 3.2. Carvedilol Alone and Associated with VAL Prevented and Reversed the Alterations in GSH and Lipid Peroxidation Induced by LDX in the Prefrontal Cortex and Striatum of Rats

Two-way ANOVA of GSH levels revealed in the PFC a significant interaction between “treatment protocol” and “experimental group” [*F*_6,83_ = 5.95, *P* < 0.0001] with significant main effect of “treatment protocol” [*F*_1,83_ = 13.97, *P* = 0.0003] and “experimental group” [*F*_6,83_ = 4.99, *P* = 0.0002]. In the ST a significant interaction between factors [*F*_6,98_ = 2.68, *P* = 0.0188] with significant main effect of “treatment protocol” [*F*_1,98_ = 13.73, *P* = 0.0003] and “experimental group” [*F*_6,98_ = 8.34, *P* < 0.0001] was also observed. In relation to MDA levels in the PFC a significant interaction between “treatment protocol” and “experimental group” [*F*_6,70_ = 3.58, *P* = 0.0038] with significant main effect of “treatment protocol” [*F*_1,70_ = 19.21, *P* < 0.0001] and “experimental group” [*F*_6,70_ = 18.67, *P* < 0.0001] was observed. In the ST there was no significant interaction between factors [*F*_6,92_ = 1.26, *P* = 0.2841], but significant main effects of “treatment protocol” [*F*_1,92_ = 10.69, *P* = 0.0015] and “experimental group” [*F*_6,92_ = 18.64, *P* < 0.0001] were observed.

**Figure 4:**
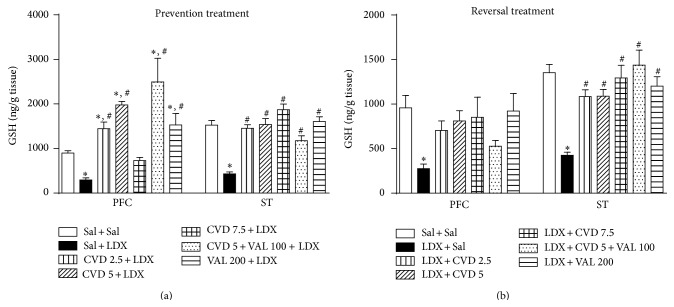
Levels of reduced glutathione (GSH) in the prefrontal cortex (PFC) and striatum (ST) of mice submitted to the prevention (a) and reversal (b) treatments. Data were analyzed by one-way ANOVA followed by Student-Newman-Keuls post hoc test. Values are expressed as mean ± SEM (6–8 animals/group). ^∗^ *P* < 0.05 versus Sal + Sal; ^#^ *P* < 0.05 versus Sal + LDX or LDX + LDX. CVD: carvedilol; LDX: lisdexamfetamine; Sal: saline; VAL: valproate.

**Figure 5:**
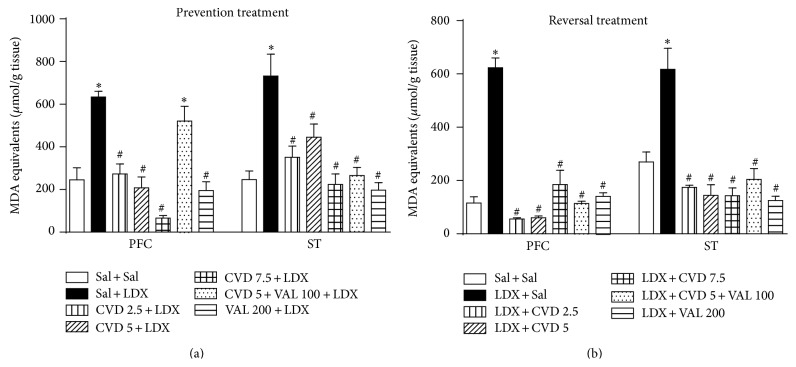
Levels of malondialdehyde (MDA), a lipid peroxidation marker, in the prefrontal cortex (PFC) and striatum (ST) of mice submitted to the prevention (a) and reversal (b) treatments. Data were analyzed by one-way ANOVA followed by Student-Newman-Keuls post hoc test. Values are expressed as mean ± SEM (6–8 animals/group). ^∗^ *P* < 0.05 versus Sal + Sal; ^#^ *P* < 0.05 versus Sal + LDX or LDX + LDX. CVD: carvedilol; LDX: lisdexamfetamine; Sal: saline; VAL: valproate.

Post hoc test showed that the administration of LDX in both prevention and reversal treatments significantly decreased the levels of GSH (Figure 4) as well as increased MDA levels (Figure 5) in the PFC and ST when compared to control (Sal + Sal) rats, as expected for an animal model of mania.

Regarding GSH levels, in the prevention treatment, the administration of CVD 2.5, CVD 5, CVD5 + VAL100, and VAL200 not only significantly prevented the decrement in GSH levels induced by LDX (*P* < 0.001) in the PFC (Figure 4(a)) but also significantly increased GSH levels in these experimental groups in relation to control animals (*P* < 0.001). In the ST all treatments prevented the decrease in GSH induced by LDX (*P* < 0.001). In the reversal treatment only VAL200 prevented the decrease in GSH levels induced by LDX in the PFC. In the ST CVD2.5, CVD5, CVD5 + VAL100, and VAL200 reversed the GSH decrement induced by LDX (*P* < 0.001) (Figure 4(b)).

The evaluation of lipid peroxidation revealed that in the PFC and ST of the animals subjected to the prevention (Figure 5(a)) and reversal (Figure 5(b)) treatments both doses of CVD and VAL200 and the association of CVD + VAL100 prevented and reversed the alterations induced by LDX (*P* < 0.001). The exception was the PFC of the rats administered CVD5 + VAL100 in which the alterations in MDA levels were not prevented by this treatment.

### 3.3. Carvedilol Alone and Associated with VAL Prevents and Reverses the Alterations in the Hippocampal Levels of BDNF Induced by LDX

**Figure 6:**
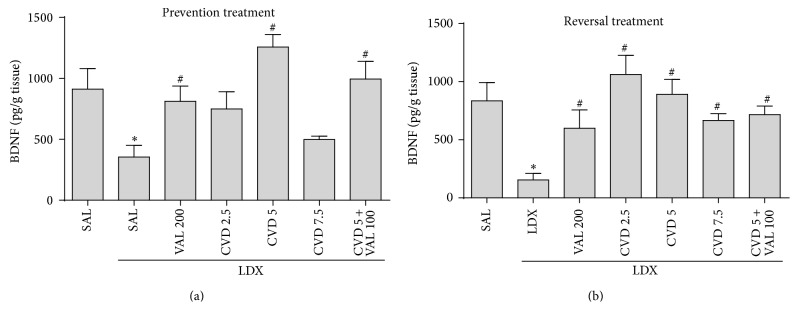
Levels of brain derived neurotrophic factor (BDNF) in the hippocampus of rats submitted to the prevention (a) and reversal (b) treatments. Data were analyzed by one-way ANOVA followed by Student-Newman-Keuls post hoc test. Values are expressed as mean ± SEM (6–8 animals/group). ^∗^ *P* < 0.05 versus Sal + Sal; ^#^ *P* < 0.05 versus Sal + LDX or LDX + LDX. CVD: carvedilol; LDX: lisdexamfetamine; Sal: saline; VAL: valproate.

Two-way ANOVA of hippocampal BDNF levels revealed no significant interaction between factors [*F*_6,62_ = 1.93, *P* = 0.0905], but main effect of “experimental group” [*F*_6,62_ = 7.48, *P* < 0.0001] was observed. BDNF is being considered a potential candidate as a biomarker for bipolar disorder [1]. In this context, as observed in Figure 6, post hoc analysis showed that the administration of LDX decreased BDNF levels in the hippocampus of rats that underwent the prevention and reversal treatments. In the prevention treatment CVD5, CVD5 + VAL100, and VAL200 significantly prevented the decrement in BDNF levels induced by LDX (*P* < 0.05). In the reversal treatment both doses of CVD as well as CVD5 + VAL100 and VAL200 reversed the decrease in these neurotrophin levels induced by LDX (*P* < 0.01).

## 4. Discussion

The results of the present work demonstrated, to the best of our knowledge, for the first time that CVD is effective in the prevention and reversal of LDX-induced mania-like behavioral and neurochemical alterations. Additionally, CVD potentiated the effects of the mood-stabilizing VAL.

Manic states include complex and multifaceted symptoms such as overactivity, hypersexuality, irritability, and reduced need for sleep [29]. To date, the evaluation of these symptoms in preclinical models of mania is almost restricted to the observation of hyperlocomotion; nevertheless this was recently criticized [12]. Thus, in our study we decided to evaluate the social interaction of the animals since this feature is increased in mania [28].

Based on our behavioral results we observed that the administration of CVD 5 mg/kg, VAL 200 mg/kg, and the combination of VAL 100 mg/kg + CVD 5 mg/kg prevented and reversed hyperlocomotion and the increased sociability induced by LDX, with the exception of VAL alone that was not able to reverse the LDX-induced alteration in social interaction. Regarding the mood-stabilizing drugs, when we first proposed this LDX-induced model of mania [9] lithium was the only drug used to assess the predictive validity of the model. Based on a previous preclinical study that demonstrated a potentiation of anticonvulsant effects by the administration of VAL 100 mg/kg and CVD 5 mg/kg, we decided to use, in the present study, VAL as mood-stabilizing drug [30].

The behavioral alterations observed in BD patients are linked to central mechanisms, such as the following [7, 31]: (i) alterations in monoamine levels, for example, the dopamine dysregulation syndrome; (ii) mitochondrial dysregulation; (iii) alterations in calcium homeostasis; (iv) neuroinflammation; (v) oxidative imbalance; and (vi) dysregulation in neurotrophin's levels. These mechanisms are also involved in the neuroprogression of this mental disorder [32], emerging, mainly the last five, as important targets for BD treatment [1, 33].

Focusing on the aforementioned targets for BD treatment, CVD is a drug that presents antioxidant and mitochondrial stabilizing properties as well as regulating intracellular calcium that may be considered as an important option for the treatment of BD. Additionally, in the last ten years the neuroprotective properties of this drug began to be studied although with incipient findings to date [34, 35].

As observed in our results CVD in addition to regulating oxidative imbalance also increased the levels of BDNF when administered alone and when combined with VAL. In fact when GSH levels are reduced there is an increased potential for cellular oxidative stress, characterized by an increase and accumulation of ROS. Furthermore, the decreased levels of antioxidants or increased levels of free radicals are related to chain reactions, such as lipid peroxidation, leading to cellular dysfunction [36]. Overall glutathione antioxidant system and lipid peroxidation are altered in animal models of mania [8, 9] as well as in BD patients [37, 38]. Oxidative imbalance leads to reduced expression of BDNF. The mechanisms linking oxidative stress to decreased BDNF levels involve several factors such as the following: (i) decrease of DNA-binding activities of activator protein-1 and cAMP response element-binding protein (CREB), BDNF transcription factor, which is associated with reduction of BDNF gene expression [39], and (ii) energy imbalance which causes a dysfunction in the N-methyl-D-aspartate (NMDA) channel resulting in decreases in BDNF gene expression [40].

Regarding BDNF, the serum levels of this neurotrophin in BD patients are decreased in depressive and manic episodes, returning to normal levels in euthymia [41] with a similar pattern of alteration in animal models of mania [9, 14]. Together, these mechanisms have been extensively implicated in the pathophysiology of schizophrenia and BD [37, 42].

Thus, based on the importance of oxidative imbalance in the pathophysiology of BD, an increasing number of preclinical [8] and clinical [43] studies are proposing antioxidants as adjunct therapies for BD. Interestingly, it was recently proposed in BD patients with medical comorbidities, such as cardiovascular and endocrine conditions, that the use of the antioxidant N-acetyl cysteine improved functional outcomes [43]. Therefore, this adds further evidences for the importance of CVD, a drug that treats cardiovascular conditions besides being antioxidant, in BD treatment.

Of note, the use, in the present study, of the association of half the dose of VAL (100 mg/Kg, twice a day) with CVD 5 maintained the effect of VAL 100 in a similar pattern to the one presented by VAL 200 mg/kg twice a day. One exception was noticed in the social interaction test where in the reversal protocol the association reversed the alteration induced by LDX whereas VAL alone did not. This is an important finding because VAL presents important side effects, such as tremor, weight gain, alopecia, more frequent sedation, and infection [44] that could be alleviated by the use of lower doses. Indeed a previous preclinical study suggested that CVD potentiates the anticonvulsant activity of VAL possibly by a pharmacodynamics interaction [30]. In this previous study the association of CVD 5 + VAL 100 was the best for the increase in seizure threshold induced by pentylenetetrazole, an effect that was accompanied by increase in GSH levels and decrease in lipid peroxidation [30].

The combination CVD 5 + VAL 100 did not prevent LDX-induced rearing alteration. This result does not compromise the antimanic-like effect of this combination observed in this study, since the parameter number of crossings is the most reliable [45, 46]. On the contrary, CVD 7.5 did not prevent LDX-induced alterations in the number of crossings, social interaction, GSH levels in the PFC, and BDNF. One possible explanation in that when compared to humans on an mg/m^2^ basis, 7.5 mg/kg CVD in rats corresponds approximately to 75 mg/daily CVD in humans. Of note, the total daily doses of 6.25 to 50 mg CVD are the most prescribed for cardiovascular conditions [47]. Thus, based on this evidence possibly 7.5 mg/kg CVD in rats was an excessive dose.

Our study has some limitations: first, animal models in psychiatry are fair representations of real complexity of the disorders. That is to say, clinical studies are necessary before we can conclude that CVD could bring tangible benefits to BD patients. Furthermore, our study evaluated a correlation between intervention and outcome and was not equipped to parse possible mechanistic pathways. Therefore, we could not establish the real mechanisms that are necessary and adequate to explain the behavioral effects of CVD; for example, mitochondrial protection by CVD was not evaluated here.

In conclusion, CVD was able to prevent and reverse oxidative imbalance and BDNF levels in the model of mania induced by LDX. Overall, the results presented here give the first preclinical evidences for the future design of clinical trials investigating the use of CVD in mania.
